# Impact of a Student-Led Research Methodology Course on Self-Perceived Research Competencies: A Quasi-experimental Study Among Medical Students in Panama, 2024

**DOI:** 10.7759/cureus.112679

**Published:** 2026-07-14

**Authors:** Roberto Sáenz, David Cedeno-De Leon, Paula Palacios-Zimmermann, Pablo Vega-Medina, Americo Rengifo, Kathiuska Bucktron, Lorena Noriega-Aguirre

**Affiliations:** 1 Medicine, Ciudad de la Salud, Caja de Seguro Social (CSS), Panama City, PAN; 2 Clinical Research Center, School of Medicine, University of Panama, Panama City, PAN; 3 Medicine, Santo Tomás Hospital, Ministry of Health (MINSA), Panama City, PAN; 4 Pulmonology, Ciudad de la Salud, CSS, Panama City, PAN

**Keywords:** latin america, medical students’ perception, panama, professional competence, research methodology, research methodology course, research on medical education, self-perceived learning, training programs

## Abstract

Introduction: Undergraduate research training improves critical thinking, although barriers related to mentorship and time persist. This study evaluated changes in self-perceived research competencies after a free extracurricular student-led research methodology course combining lectures, practical workshops, and mentorship.

Methodology: A single-group quasi-experimental study with a retrospective pretest-posttest design was conducted among medical students after completion of this 13-session, 30-hour extracurricular course. Descriptive statistics were used to summarize outcomes; pre- and post-course self-perceived competencies were compared using the Wilcoxon signed-rank test; grades were modeled according to attendance using linear regression; and perceived barriers were explored.

Results: Among 59 participants (mean age 23.7 ± 1.58 years; 36, 61.0%, women), those feeling *well *or *very well prepared *increased from 8 (13.6%) to 37 (62.7%). Attendance was positively associated with grade (*r* = 0.4716; β = +2.39 points per session attended; *R*² = 22.2%; *P* = 0.00016). The Wilcoxon test showed a significant difference (statistic = 0.0; *P* < 0.000001).

Conclusions: A structured student-led course was associated with higher self-perceived research competencies and better academic performance. Incorporating mentorship and practical sessions is recommended to strengthen outcomes and support implementation in other faculties.

## Introduction

Research training during medical school is essential for developing critical thinking and evidence-based clinical practice. The main barriers described include a lack of mentors, limited time, and insufficient institutional guidance toward scientific activity [1,2]. However, early participation in research projects is essential, as it is associated with an almost twofold higher likelihood of continuing scientific activities during medical school [3].

In Latin America, it has been reported that fewer than 10% of undergraduate programs in the region include structured research training and that lack of mentorship and funding represent relevant limitations [4].

In contrast, interventions with positive effects have also been studied. In a study across 19 countries, membership in student scientific societies was associated with a higher likelihood of publication (odds ratio (OR) = 2.09; 95% confidence interval (CI): 1.41-3.10), as was training in scientific writing (OR = 1.98; 95% CI: 1.30-3.00) [5].

A virtual research course reported a 35% increase in self-confidence for critical appraisal and study design [6]. Similarly, an in-person workshop increased knowledge from 48% to 53.9% and intention to pursue a research career from 75% to 90.7% [7].

Student-organized research courses have been evaluated in various studies, showing increases in self-efficacy (+0.62 points on a Likert scale), perceived need for competencies (+0.54 points), and research interest (+0.49 points; *P* < 0.01) [8]. However, evidence on student-led extracurricular research methodology courses in Latin American medical schools remains limited, particularly in Panama, where the association between participation in this type of program and self-perceived research competencies has not been well described.

Given that the intervention had already been implemented as an extracurricular educational activity and that a prospective baseline assessment was not available, a single-group quasi-experimental study with a retrospective pretest-posttest design was selected as a pragmatic approach to evaluate perceived changes after the course. The primary objective of this study was to evaluate changes in self-perceived research competencies associated with participation in a student-organized research methodology course among medical students in Panama. Secondary objectives were to describe course satisfaction, academic outputs, perceived barriers to conducting research, and the association between attendance and final grade.

A preliminary version of this work was presented at the 2025 National Congress of the Panama Medical Association (Colegio Médico de Panamá).

## Materials and methods

Study design

A single-group quasi-experimental before-and-after study using a retrospective pretest-posttest design was conducted. This retrospective pretest-posttest design was used to estimate perceived changes after the intervention without influencing participants' initial responses or requiring a formal baseline assessment. This approach was selected to reduce response-shift bias associated with prospective baseline measurements in educational settings.

Population and sample

The target population included all students enrolled in a student-organized extracurricular course on clinical research methodology. Participants were from different years of the medical program. The course was an extracurricular, non-mandatory educational activity designed to provide additional training in clinical research methodology and complement the formal research and biostatistics content included in the institutional medical curriculum. The sample consisted of 59 students enrolled in the course, all of whom completed the final self-perception questionnaire administered at the end of the course, with no missing data. A formal sample size calculation was not performed because all enrolled students who completed the final questionnaire were included, constituting a convenience sample.

Intervention

The 2024 Research Methodology Course was an academic activity organized by the Scientific Committee of the University of Panama Medical Students’ Association (CCAEMP), with endorsement from the Panama Medical Association and the Clinical Research Center of the School of Medicine, University of Panama (Centro de Investigaciones Clínicas, CICLI-UP). The intervention consisted of a free educational program comprising 13 sessions delivered between February 29 and May 30, 2024, aimed at medical students interested in developing competencies in clinical research. 

The course included 30 academic hours, divided into 14 in-person hours and 16 asynchronous hours, with sessions delivered by physicians with national and international experience in clinical research. The teaching strategy combined lectures, practical workshops, and discussion periods delivered both in person and virtually. Attendance was monitored using signed attendance sheets during in-person sessions and periodic screenshots during virtual sessions. 

To receive the course certificate, students were required to meet three course completion criteria defined by the authors, who were also the course organizers: active participation in at least 80% of sessions, passing two midterm exams developed by the instructors, and submission of an original research protocol evaluated by committee members and invited faculty. These criteria were established for the academic administration of the course and were not adapted from external copyrighted criteria or scoring systems. The final grade analyzed in this study was calculated exclusively as the average score of the two midterm exams. Attendance and submission of the research protocol were course completion and certification criteria, but they were not included as components of the final grade used in the analysis. Content was organized progressively, from foundational methodological concepts to more advanced tools such as statistical analysis and scientific publication. The session schedule is presented in Table 1.

**Table 1:** Academic schedule. *Epi Info is a free epidemiologic software package developed by the Centers for Disease Control and Prevention; it was included in the course because of its accessibility for students and public health researchers. PICO, population-intervention-comparison-outcome

Session	Content
First session	PICO question, study design, objectives, and study title (1 hour)
Second session	Advanced searching in PubMed and Google Scholar (1 hour)
Third session	Critical appraisal of scientific articles (1 hour)
Fourth session	Problem statement, justification, and theoretical framework (1 hour)
Fifth session	Study designs: classification and limitations (1 hour)
Sixth session	Sample size calculation and sampling techniques (1 hour)
Seventh session	Data collection instruments and informed consent (1 hour)
Eighth session	Submission process to a bioethics committee (1 hour)
Ninth session	Data analysis with Epi Info* (1 hour)
Tenth session	Data collection processes as a student (retrospective/prospective) (1 hour)
Eleventh session	How to publish your first scientific article (1 hour)
Twelfth session	Survival studies and Kaplan-Meier plots (1 hour)
Thirteenth session	How to respond during the editorial process (2 hours)

Procedures

Data were collected using a structured digital questionnaire created by the authors for this study. The instrument included domains adapted from prior studies on medical student research participation, barriers to research, research training, self-perceived research competencies, and student-engaged research curricula [1,2,6,8]. No questionnaire items were directly copied from previously published instruments. The questionnaire was reviewed by advisors with expertise in clinical research methodology to assess content relevance, clarity, and alignment with the study objectives. Before administration, the questionnaire was pilot tested for clarity and comprehension among medical students who were not included in the final analysis. No formal psychometric validation, construct validity assessment, or reliability testing was performed. The questionnaire captured variables of interest, including demographic characteristics, course participation, final grade, self-perceived research competency before and after the course, course satisfaction, academic outputs, perceived barriers to conducting research, and perceived useful or improvable aspects of the course.

The questionnaire was administered after the course had concluded to assess perceived changes in knowledge and competencies. The primary outcome was self-perceived research competency, measured using a five-category ordinal scale: not prepared at all, slightly prepared, moderately prepared, well prepared, and very well prepared. Secondary variables included course attendance, final grade, satisfaction with the activity, completion of a research protocol, presentation of the research project, and perceived barriers to conducting research. The questionnaire was reviewed by faculty with experience in clinical research for clarity and content relevance. The complete English-language data collection instrument is provided in Appendix A.

Data analysis

Statistical analyses were performed using jamovi, version 2.6.44 (The jamovi project, Sydney, Australia). Continuous variables were summarized using means and standard deviations, and categorical variables using absolute and relative frequencies. Changes in perceived competency before and after the course were evaluated using the Wilcoxon signed-rank test after non-normality was confirmed with the Shapiro-Wilk test. The relationship between the number of sessions attended and the final grade was assessed using Pearson’s correlation coefficient and a simple linear regression model, estimating β coefficients with 95% CIs. The association between perceived competency and final grade was evaluated using Spearman’s correlation coefficient. A two-sided *P*-value < 0.05 was considered statistically significant for all analyses. No qualitative data collection or formal qualitative analysis was performed; perceived barriers and course-related aspects were assessed using predefined response options and summarized descriptively. This manuscript was reported using the TREND (Transparent Reporting of Evaluations with Nonrandomized Designs) guidance for nonrandomized evaluations, with items not applicable to a single-group educational intervention marked as not applicable in the checklist [9]. The completed TREND checklist is provided in Appendix B.

Ethical considerations

The study received endorsement from the School of Medicine, University of Panama, and approval from the Panama Clinic Bioethics Committee (CBI-TPC-069-2023). Written informed consent was obtained electronically from all participants before completion of the study questionnaire.

## Results

The sample consisted of 59 students, with a mean age of 23.7 years (standard deviation (SD) = 1.58); 36, 61.0%, were women. The mean course grade was 83.8 (SD = 12.6), with a mean attendance of 11.9 sessions (SD = 2.49).

In the self-perception assessment, the number of students who considered themselves *well prepared* or *very well prepared* increased from 8 of 59 (13.6%) before the course to 37 of 59 (62.7%) after the course. The proportion of participants who were *satisfied *or *completely satisfied* with the activity was 51 of 59 (86.4%), and 58 of 59 (98.3%) reported that they would recommend the course. A total of 38 of 59 (64.4%) students developed a research protocol, and 39 of 59 (66.1%) presented their project at a regular meeting (Table 2).

**Table 2:** Perceived research competencies, satisfaction, and academic outcomes of the course. Values are presented as absolute frequency and percentage, *n* (%). Values of 0 (0%) indicate categories that were assessed but not selected by any participant. A single hyphen (-) indicates that the item was not applicable or was not assessed at that time point. Total sample size: *N* = 59.

Variable	Category	Before the course, *n* (%)	After the course, *n* (%)
Perceived research competency	Not prepared at all	9 (15.3%)	0 (0%)
	Slightly prepared	29 (49.2%)	0 (0%)
	Moderately prepared	13 (22.0%)	22 (37.3%)
	Well prepared	4 (6.8%)	29 (49.2%)
	Very well prepared	4 (6.8%)	8 (13.6%)
Course satisfaction level	Moderately satisfied	-	8 (13.6%)
	Satisfied	-	36 (61.0%)
	Completely satisfied	-	15 (25.4%)
Would recommend the course	Yes	-	58 (98.3%)
	No	-	1 (1.7%)
Completed a research protocol	Yes	-	38 (64.4%)
	No	-	21 (35.6%)
Presented the project at a regular meeting	Yes	-	39 (66.1%)
	No	-	20 (33.9%)

The most frequent barriers included a lack of research culture in 35 (59.3%) participants, limited methodological knowledge in 34 (57.6%), and the perception of research as a complex field in 31 (52.5%) (Table 3).

**Table 3:** Perceived barriers to conducting research among medical students. Data are presented as numbers and percentages of participants (*N* = 59). Participants could select multiple responses; therefore, percentages may sum to more than 100%.

Barrier	Frequency (*N* = 59)	Percentage
Lack of a research culture	35	59.3%
Limited knowledge of research methods	34	57.6%
Perception of research as a challenging field	31	52.5%
Institutional barriers to initiating research projects	22	37.3%
Difficulty in data collection	20	33.9%
Other	5	8.5%

Participants identified invited speakers, practical sessions, and mentorship as the most positive aspects of the course. Inferential analysis showed a significant positive association between session attendance and final grades, as well as a significant improvement in self-perceived research competencies from baseline to post-course assessment (Table 4).

**Table 4:** Participant course preferences and inferential analysis of research competencies. Statistical significance was defined as *P* < 0.05. CI, confidence interval; *r*, Pearson correlation coefficient; rho, Spearman’s rank correlation coefficient; β, unstandardized regression coefficient; *R*², coefficient of determination; stat, Wilcoxon signed-rank test statistic

Variable	Metric/Category	Value (*N* = 59)
Course aspects most liked	Invited speakers	43 (72.9%)
	Practical sessions	40 (67.8%)
	Mentorship	32 (54.2%)
Attendance vs. Final grade	Pearson r (95% CI)	0.47 (0.25-0.65)
	Linear Regression β (95% CI)	2.39 (1.18-3.60)
	Coefficient of determination (*R*^2^)	22.2%
	*P*-value	<0.001
Competency vs. Final grade	Baseline competency (rho; *P*-value)	0.10 (0.460)
	Post-course competency (rho; *P*-value)	0.33 (0.011)
Competency improvement	Wilcoxon signed-rank test (stat; *P*-value)	0.0 (<0.001)

**Figure 1:**
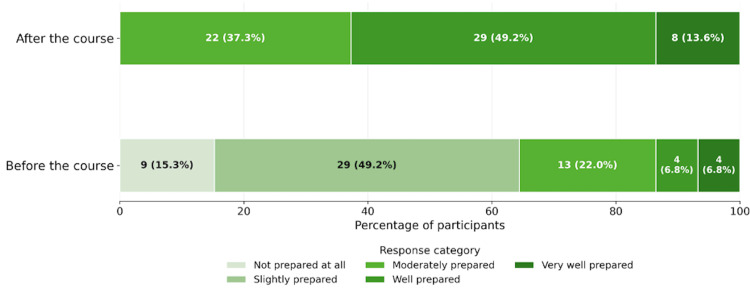
Perceived research competency before and after the course. Percentage distribution of self-perceived research competency before and after the course among medical students who completed the questionnaire. Values within the bars are presented as *n* (%); *N* = 59. The Wilcoxon signed-rank test showed a statistically significant difference between pre-course and post-course self-perceived competency (*P* < 0.000001). Image created by the authors using Microsoft Excel (Microsoft Corporation, Redmond, WA).

The Wilcoxon signed-rank test comparing perceived competency before and after the course showed a statistically significant difference (Wilcoxon signed-rank test statistic = 0.0, *P* < 0.000001) (Figure 1).

## Discussion

Research training during undergraduate medical education continues to face persistent limitations, particularly in settings where research is not systematically integrated into the curriculum [1]. In this context, the present study found that participation in an extracurricular course was associated with higher self-perceived research competency, highlighting the potential of student-led initiatives as complementary training strategies. Because the primary outcome was based on students’ self-perception, these findings should be interpreted as changes in perceived competency rather than direct evidence of improved objective research knowledge or performance. These findings suggest that this type of intervention may play a relevant role in early exposure to research and underscore the need to reflect on the integration of structured research training experiences within medical schools.

Several studies have shown that extracurricular courses can contribute to strengthening academic research competencies during undergraduate medical training. In a training program for health sciences students, Alnajjar et al. reported significant improvements in research knowledge and attitudes following a structured extracurricular course. They also identified gaps in students' baseline knowledge before the intervention, highlighting the potential of this type of program as an early-entry strategy into research [10]. Internationally, institutionalized programs at universities such as Duke and Stanford have integrated research as a structural component of the medical curriculum and have been associated with a greater orientation toward academic trajectories and scientific productivity during training [11]. Although the institutional context of the present study differs from these models, our findings suggest that well-structured extracurricular initiatives may be associated with improved self-perceived research competency, even in resource-limited settings.

The observed change in self-perceived research competency may be explained by the convergence of several pedagogical factors, including accessible instructors, mentorship support, and the program’s participatory structure. Evidence indicates that academic mentorship is associated with the development of research skills and academic performance, particularly when implemented in a structured and sustained manner. Empirical studies have shown that faculty mentoring can directly influence students’ acquisition of research competencies. At the same time, formal mentor training improves the quality of the mentoring process, indirectly strengthening educational outcomes among students engaged in research activities [12,13].

The association between attendance and academic performance suggests that sustained participation reinforces learning, consistent with educational models that emphasize active participation and tutoring as facilitators of academic achievement [14]. Because attendance was not incorporated into the final grade, this association was not due to direct circularity between attendance and grading. However, it should still be interpreted cautiously, as higher attendance may reflect greater motivation, availability, or engagement with the course.

Persistent barriers to conducting research

Despite the improvement in self-perceived research competencies associated with the course, barriers that limit translating these improvements into effective project execution persist. This pattern suggests that methodological training may primarily affect the individual level without substantially modifying structural constraints within the academic environment [15]. Prior evidence indicates that student participation in research remains limited by factors such as lack of mentorship, limited protected time, and insufficient institutional support, even in settings where formal training in methodology exists [1,2]. In Latin America, it has been reported that only a small proportion of students achieve basic scientific competencies and that affiliation with student scientific societies or research groups is associated with a higher likelihood of scientific output, with adjusted prevalence ratios greater than 3 [5].

Overall, these data suggest that time-limited educational interventions tend to strengthen theoretical knowledge and perceived competency but have a constrained impact on applied skill development in the absence of ongoing mentorship and sustained practical experiences [6-8]. In contrast, research programs integrated longitudinally, with structured supervision and prolonged exposure, have been associated with higher academic satisfaction and a greater likelihood of considering long-term research pathways, such as pursuing doctoral studies [3].

Limitations

This study has limitations related to sample size and the inclusion of a single institution, which restricts the generalizability of the results. The use of a retrospective pretest-posttest design may have introduced recall bias, social desirability bias, and response-shift bias. Although the questionnaire was administered within two weeks after course completion, participants may not have accurately reconstructed their baseline level of preparedness because no prospective baseline measurement was obtained before the intervention. Social desirability bias may also have influenced participants to report more favorable post-course perceptions, particularly because the intervention was positively received. In addition, response-shift bias may have occurred if students’ understanding of research competencies changed after completing the course, leading them to reassess their baseline preparedness differently from how they would have rated it before the intervention. These biases may have contributed to the magnitude of the observed improvement in self-perceived competency. In addition, the questionnaire was developed specifically for this study. It was reviewed for content relevance and clarity, but it did not undergo formal psychometric validation or reliability testing, which may limit confidence in the precision of the self-perceived competency measurement. Finally, the absence of a control group and objective measures of research knowledge or performance limits the ability to determine whether perceived improvements reflect actual competency gains.

## Conclusions

An extracurricular, student-led research methodology course was associated with improved self-perceived research competency among medical students; however, this finding should not be interpreted as direct evidence of objective improvement in research knowledge or performance. After the course, more participants considered themselves well or very well prepared to conduct research, and most reported high satisfaction and willingness to recommend the activity. The course also supported academic outputs, including research protocol completion and student project presentations. The positive association between attendance and final grade suggests that sustained participation may reinforce learning. Although barriers such as limited research culture and insufficient methodological knowledge remained common, these findings support accessible, practical, mentorship-centered training as a feasible strategy to promote early research engagement. Future programs should strengthen longitudinal mentorship and institutional support to translate perceived competency improvements into sustained research participation.

## Appendices

Appendix A 

**Table 5:** Data Collection Instrument in English For multiple-selection questions, participants could select all options that applied. The questionnaire was created by the authors for this study, with domains adapted from prior studies on medical student research participation, barriers to research, research training, self-perceived research competencies, and student-engaged research curricula [1,2,6,8]. No questionnaire items were copied verbatim from previously published instruments.

Question	Response options
Age	Numeric response
Gender	Categorical response
Semester	Numeric or categorical response
Research area	Open-ended response
Did you participate in the research methods course?	Yes / No
Number of sessions attended	Numeric response
Average final grade	Numeric response
Before the course, how prepared did you perceive yourself to be in research competencies?	Five-point Likert scale: one = not prepared at all; two = slightly prepared; three = moderately prepared; four = well prepared; five = very well prepared
After the course, how prepared did you perceive yourself to be in research competencies?	Five-point Likert scale: one = not prepared at all; two = slightly prepared; three = moderately prepared; four = well prepared; five = very well prepared
How satisfied are you with the course?	Five-point Likert scale: one = not satisfied at all; two = slightly satisfied; three = moderately satisfied; four = satisfied; five = completely satisfied
Would you recommend the course to other students?	Yes / No
Did you complete a research protocol?	Yes / No
Did you present your research project at a regular meeting?	Yes / No
What barriers to conducting research do you identify?	Multiple selection: lack of a research culture; limited knowledge of research methods; institutional barriers to initiating research projects; difficulty in data collection; perceiving research as a challenging field; other
Which aspects of the course do you consider most useful or in need of improvement?	Multiple selection: interaction and group work; practical sessions; discussion sessions; mentors; invited speakers; periodic evaluations; other

Appendix B

**Table 6:** TREND Checklist for Nonrandomized Evaluations TREND: Transparent Reporting of Evaluations with Nonrandomized Designs. Items not applicable to a single-group educational intervention were marked as not applicable. Applied for manuscript: Impact of a Student-Led Research Methodology Course on Self-Perceived Research Competencies: A Quasi-Experimental Study Among Medical Students in Panama, 2024

Item No.	Section/topic	TREND recommendation	Manuscript section
1	Title and abstract	Provide information on allocation to interventions, use a structured abstract, and describe the target population or study sample.	Title; Abstract. Allocation to study conditions was not applicable because this was a single-group intervention study.
2	Background	Explain the scientific background and rationale for the study, including theoretical or educational rationale when applicable.	Introduction
3	Participants	Describe eligibility criteria, recruitment method, recruitment setting, and settings or locations where data were collected.	Materials and Methods – Population and sample; Procedures
4	Intervention	Describe the intervention, including content, delivery method, unit of delivery, deliverers, setting, exposure quantity and duration, time span, and adherence-related activities.	Materials and Methods – Intervention; Table 1
5	Objectives	State the specific objectives and hypotheses, when applicable.	Introduction
6	Outcomes	Clearly define primary and secondary outcome measures, data collection methods, measurement quality procedures, and information on instrument validity or reliability when available.	Materials and Methods – Procedures; Appendix 2; Limitations
7	Sample size	Explain how the study size was determined and whether any interim analyses or stopping rules were used.	Materials and Methods – Population and sample
8	Assignment method	Describe the unit of assignment, method used to assign units to study conditions, and methods used to minimize bias from nonrandomization.	Not applicable. This was a single-group quasi-experimental study without assignment to comparison conditions.
9	Blinding/masking	State whether participants, intervention deliverers, and outcome assessors were blinded to study condition assignment, and describe how blinding was implemented or assessed.	Not applicable. Blinding was not feasible because this was an educational intervention with a single study group.
10	Unit of analysis	Describe the smallest unit analyzed and whether it differed from the unit of assignment.	Materials and Methods – Data analysis. The unit of analysis was the individual participant.
11	Statistical methods	Describe statistical methods for primary outcomes and additional analyses, methods for missing data if used, and statistical software.	Materials and Methods – Data analysis
12	Participant flow	Describe participant flow through enrollment, exposure to the intervention, follow-up, and analysis; report protocol deviations when applicable.	Results. All enrolled students who completed the final questionnaire were included in the analysis.
13	Recruitment	Report dates defining recruitment, intervention, and follow-up periods.	Materials and Methods – Intervention; Procedures
14	Baseline data	Report baseline demographic characteristics and relevant baseline characteristics of participants; describe comparisons between retained and lost participants when applicable.	Results
15	Baseline equivalence	Report baseline equivalence between study groups and statistical methods used to control for baseline differences.	Not applicable. This was a single-group study without comparison groups.
16	Numbers analyzed	Report the number of participants included in each analysis and provide absolute numbers when feasible.	Results; Tables 2-4; Figure 1
17	Outcomes and estimation	Summarize primary and secondary outcomes for each study condition, include estimates and precision when applicable, and report null or negative findings.	Results; Tables 2-4; Figure 1
18	Ancillary analyses	Summarize other analyses performed, indicating whether they were prespecified or exploratory.	Results
19	Adverse events	Summarize important adverse events or unintended effects in each study condition.	Not applicable. No adverse events were expected or assessed for this educational intervention.
20	Interpretation	Interpret results considering objectives, potential bias, imprecision, multiple analyses, limitations, mechanisms of action, implementation barriers, and implications.	Discussion; Limitations
21	Generalizability	Discuss external validity considering the study population, intervention characteristics, setting, follow-up, adherence, and contextual factors.	Discussion; Limitations
22	Overall evidence	Interpret the results in the context of current evidence and relevant theory.	Discussion
